# Editorial: Insights in coronary artery disease: 2022

**DOI:** 10.3389/fcvm.2023.1269388

**Published:** 2023-09-07

**Authors:** Turgay Celik

**Affiliations:** Cardiovascular Section, Department of Internal Medicine, Wake Forest School of Medicine, Winston-Salem, NC, United States

**Keywords:** coronary flow reserve, coronary arterial lesions, OCT, IVUS (intravascular ultrasound), coronary artery revascularization interventions

**Editorial on the Research Topic** Insights in coronary artery disease: 2022

Atherosclerosis is a multifactorial disease that starts in childhood; inflammation plays a role at every step of atherogenesis, and risk factors further accelerate the underlying inflammatory process (1). Atherosclerotic disease affects the entire vessel and may cause difficulties in determining the significance of the lesion by angiography. Serial lesions can be a diagnostic challenge, as measuring the level of stenosis using invasive tests is affected by the complexity of factors (2). The combination of two lipid measures as the ratio of TG to HDL-C has been shown to be a reliable marker of endothelial damage and atherosclerosis caused by metabolic syndrome and insulin resistance. In this context, a high TG/HDL-C ratio, proposed as a marker of atherogenic dyslipidemia, has been associated with adverse long-term cardiovascular outcomes (3). TG/HDL-cholesterol ratio is seen as a new risk factor in a recent START study (De Luca et al.).

An acute coronary event due to a thrombosed lesion developing after mechanical rupture of an atheroma causes sudden death. Various morphology and tissue composition factors such as the cap thickness, lipid core stiffness, remodeling index, and blood pressure play a role in its mechanical stability. More recently, the presence of microcalcifications has also been shown to be an important factor. In a well-designed study, the authors showed that microcalcifications and cap thickness are the two most alarming biomechanical traits that govern the risk of mechanical rupture (Corti et al).

It is difficult to decide on coronary revascularization in patients with chest pain and moderate coronary artery stenosis. Therefore, functional evaluation is essential besides the anatomical evaluation of the presence of coronary artery. Fractional flow reserve (FFR) has been accepted as the gold standard for functionality in avoiding unnecessary interventions in the intermediate stenoses (Lee et al.). Computed tomography angiography (CTA) is used in a quick and easy way for coronary non-invasive evaluation. Intravascular ultrasound (IVUS) is used to show the morphological features of the lesion. Optical coherence tomography (OCT) has been observed to be more effective than IVUS in accurate anatomical assessment of coronary stenotic lesions with exceptionally high resolution during angiography (Lee et al.). In addition, computational fluid dynamics (CFD) have been applied to estimate computational FFR from coronary CTA- or OCT-based three-dimensional coronary model without using additional pressure guide wires or hyperemic agents.

Percutaneous coronary intervention (PCI) has a well-established role in revascularization for CAD. Despite the advances in PCI, the pathological changes that occur within the stent still remain a mystery (Abouelnour and Gori). Today, we can evaluate the pathophysiological changes in the coronary vessels with different techniques. Many biomechanical factors, some preventable and some not, may cause restenosis after stent implantation. Intravascular imaging provides unique insights into the biological and mechanical issues that cause stent restenosis (Abouelnour and Gori).

Revascularization of chronic total occlusion (CTO) is considered a complex PCI and carries a higher risk of procedural failure, complications, and in-stent restenosis than other PCIs (4). CTO revascularization is among the most challenging procedures in interventional cardiology and no significant difference in mortality and morbidity was observed when compared with optimal medical treatment (Juricic et al.). Furthermore, comprehensive evaluation and advanced techniques are important to increase success rates and decrease complications. Intravascular examinations used during the procedure increase the success rate in this context. A recent study concluded that pre-stent IVUS assessment in CTO PCI provides important information on vessel morphology and size (Blessing et al.).

In addition to the pathophysiological evaluation before PCI, FFR is also a recommended method to increase the efficiency of revascularization (Leone et al.). A TARGET-FFR study found that FFR values of routine post-PCI physiology guidance can safely and effectively improve the final FFR values in a significant number of the worst-affected patients. FFR value ≥0.90 after PCI was shown to be associated with a reduced risk of adverse cardiovascular events (5).

In STEMI patients treated with PCI, the evaluation of coronary microcirculatory resistance index (IMR) may be a potential prognostic index in patients with multi-vessel disease (MVD) if it correlates significantly with differences in outcome and LV remodeling (6). In a recent study, Fineschi et al. (7) showed that impaired coronary microvascular function is commonly detected by IMR evaluation in the infarct territory and shows an early slow recovery over time after reperfusion. Besides, it was found that post-angioplasty IMR values were negatively correlated with LVEF only in patients with anterior MI.

The COMPARE II trial (7), a 5-year analysis comparing biodegradable polymer drug-eluting stents (BP-DES) with durable polymer DES (DP-DES) in the PCI population found similar early and late-term safety and efficacy results. However, BP-DES has been produced against persistent inflammation due to the presence of polymers, which are stated to be the most important cause of late stent thrombosis, and Lee and coworkers demonstrate that it reduces mortality due to late stent thrombosis compared with DP-DES (Lee et al.).

In conclusion, there have been important developments in the diagnosis, treatment, and follow-up of CAD. Although there are significant improvements, some essential problems remain unsolved in the management of patients with CAD.
